# Adherence to the enhanced recovery after surgery protocol and its influencing factors among patients in Southwestern China: a multicenter cross-sectional study

**DOI:** 10.3389/fmed.2025.1660083

**Published:** 2025-10-20

**Authors:** Tong Mu, Yuanhang Chen, Li Ren, Feng Lv, Jingjie Hu, Chunyuan Liu, Chaofeng Wang, Ran An, Yiwei Shen

**Affiliations:** ^1^Department of Anesthesiology, The First Affiliated Hospital of Chongqing Medical University, Chongqing, China; ^2^Department of Statistics, Faculty of Science, Chinese University of Hong Kong, Hong Kong, Hong Kong SAR, China; ^3^Department of Anesthesiology, The People’s Hospital of Liangping District of Chongqing, Chongqing, China; ^4^Department of Anesthesiology, Chongqing University Cancer Hospital, Chongqing, China

**Keywords:** adherence, inpatients, ERAS, Southwestern China, influencing factors

## Abstract

**Background:**

There is limited research on adherence to Enhanced Recovery After Surgery (ERAS) protocols among hospitalized patients and the factors influencing it, both in China and globally.

**Methods:**

A random sample of 1,203 participants from 45 hospitals in Southwestern China was surveyed on ERAS protocol implementation, including 16 items. A questionnaire assessed awareness, attitudes, and social and environmental support factors. Multivariable linear regression model was used to analyze factors affecting ERAS adherence.

**Results:**

The final analysis included 806 surgical patients, with an average ERAS adherence rate of 71.5% (56.3, 81.3%). The highest completion rates were seen in avoidance of prolonged fasting (88.6%) and prophylactic antibiotic use (88.2%), while the lowest were in preoperative oral carbohydrate intake (42.2%) and deep vein thrombosis prevention (52.4%). Factors positively influencing adherence included educational background (*p* = 0.010), surgical grade (*p* = 0.013), positive attitudes toward ERAS (*p* < 0.001), perception of ERAS (*p* < 0.001) and social and environmental supports (*p* < 0.001). Negative influences included non-tertiary center status (*p* = 0.039) and negative attitudes (*p* = 0.002).

**Conclusion:**

ERAS adherence in Southwestern China remains low, with various factors such as hospital grade, patients’ educational background, their perceptions and attitudes toward ERAS, and their social and environmental support influencing ERAS adherence rates.

**Clinical trial registration:**

http://clinicaltrials.gov, Identifier ChiCTR2400086759.

## 1 Introduction

The enhanced recovery after surgery (ERAS) pathway is a multimodal perioperative care approach that has been demonstrated to significantly improve patient outcomes. This comprehensive protocol is designed to shorten hospital stays, reduce perioperative stress and pain, decrease postoperative complications, and promote rapid patient recovery through the synergistic effects of its various components (1–3). Despite the proven benefits of ERAS protocols, which comprise several different perioperative interventions, not all participants are able to fully adhere to the prescribed regimen. The implementation and adherence to ERAS protocols are crucial for achieving optimal outcomes (4–6). However, several challenges impede successful implementation, including a lack of knowledge toward ERAS, resistance to change in established practices, and shortages of adequately trained staff (7).

A growing body of research has consistently confirmed that adherence to ERAS protocols is strongly associated with improved postoperative rehabilitation outcomes in patients undergoing various surgical procedures, including colorectal and orthopedic surgeries (8–10). Furthermore, additional studies have demonstrated that enhanced adherence to ERAS protocols leads to significant improvements in clinical rehabilitation (4, 11). Since its gradual introduction to China in 2013 and the formal establishment of the China ERAS Working Group in 2016, numerous domestic medical institutions have begun to implement ERAS protocols, recognizing their superior outcomes. Preliminary research conducted by our team has revealed that improved adherence to the ERAS protocols was associated with enhanced recovery and better patient experiences in individuals undergoing hysterectomy (10). However, a concerning finding from another multicenter survey indicated that only 14.83% of inpatients in China were able to engage in ERAS-related exercises on a daily basis (12). Therefore, these findings underscore the urgent need to identify and address the barriers hindering adherence to ERAS protocols.

While existing studies indicate that higher ERAS adherence rates are associated with reduced postoperative complications and improved recovery outcomes, comprehensive primary data regarding ERAS adherence and its influencing factors among hospitalized patients in China remain notably scarce. To address this knowledge gap, this paper presents a detailed analysis of the current profile and factors affecting ERAS adherence in Southwestern China.

## 2 Methods

### 2.1 Participants

A stratified random sampling survey was conducted, which received approval from the ethics committee of the First Affiliated Hospital of Chongqing Medical University (the organizing center) and was subsequently filed with the ethics committees of the participating centers. The study encompassed patients from 45 hospitals in Southwestern China, selected based on geological distribution, from July 15 to July 25, 2024 (participating hospitals and codes are listed in Appendix 1). Prior to patient enrollment, the study was registered with ClinicalTrials.gov (ChiCTR2400086759). Patients were informed about the ERAS protocols and provided signed informed consent before study entry. The study was conducted in strict adherence to the principles outlined in the Declaration of Helsinki.

The study population comprised inpatients from the departments of gastroenterology, gynecology, hepatobiliary surgery, and urology who underwent elective abdominal surgery and were subjected to perioperative ERAS protocols. The ERAS protocol consists of 16 items encompassing preoperative, intraoperative, and postoperative interventions, which are based on the practice guidelines (13) for surgery established by the ERAS society (Table 1). All patients received ERAS education during their admission education period, and were informed of the relevant procedures and information related to ERAS as well as the discharge criteria.

**Table 1:** Perioperative ERAS protocols (16 items).

Period	ERAS component
Pre-operative	Preoperative optimization (Smoking and alcohol consumption should be stopped 4 weeks before surgery)
	No prolonged preoperative fasting (6–8 h for solid food, 2 h for clear liquids)
	Multimodal prevention of DVT (physical prophylaxis combined with low molecular weight heparin administration)
	Preoperative antibiotic prophylaxis (Intravenous cefoxitin 1.5 g or ceftriaxone 1 g 30 min before incision)
	Preoperative carbohydrate intake (intake of 400 mL 10% glucose solution: up to 2–3 h before the induction of anesthesia)
Intra-operative	Anesthesia optimization (General anesthesia with rapid short-acting agents combined with TAP block and lung-protective ventilatory strategy)
	Minimally invasive surgery (Laparoscopic or robotic surgery)
	No drainage placed routinely
	GDFT by anesthetic team with a focus on avoiding fluid overload
	Hypothermia prevention
	PONV prevention (with >2 antiemetic agents)
Post-operative	Multimodal analgesia (PCIA, TAP, NSAIDs, COX-2 inhibitor)
	Early exercise (out-of-bed activity for 2 h on the first postoperative day and 4–6 h from the second postoperative day to discharge)
	Early oral feeding (drink water 2 h after surgery, oral nutritional supplements on the first day after surgery, semisolid diet on the second day after surgery)
	Early removal of drainage (Early removal of drainage tubes within three days after surgery and early removal of urinary catheter within 24 h)
	Nutrition support therapy

COX, cyclooxygenase; DVT, deep vein thrombosis; PONV, postoperative nausea and vomiting; PCIA, patient-controlled intravenous analgesia; TAP, transversus abdominis plane; NSAIDs, nonsteroidal anti-inflammatory drugs; GDFT, Goal-Directed Fluid Therapy.

Inclusion criteria were established as follows: (1) Inpatients from units that have undergone ERAS training and have implemented ERAS plans for elective abdominal surgery in the fields of gastroenterology, hepatobiliary surgery, gynecology, and urology; (2) Age range of 18–70 years, irrespective of gender; (3) ASA status I-III; (4) Absence of severe preoperative cardiorespiratory dysfunction; and (5) Patients undergoing general anesthesia. Exclusion criteria were defined as: (1) Emergency surgery; (2) Individuals refused to participate; (3) Individuals with cognitive dysfunction; (4) Acoustic dysfunction; (5) Visual impairment; and (6) Those who did not provide informed consent. Dropout criteria were established as: (1) Patients experiencing significant intraoperative bleeding (exceeding 2000 mL); (2) Subjects withdraw from the study; (3) Subjects withdrawing informed consent; (4) Patients undergoing unplanned secondary surgery; (5) Discontinuation due to adverse events; and (6) Loss to follow-up. Cases that seriously violated the inclusion criteria or lacked evaluable records were excluded from the study.

### 2.2 Study design

The survey was conducted under the leadership of the anesthesiology departments in participating institutions, with collaborative support from relevant surgical departments. One month prior to the survey’s commencement, investigators from each institution underwent rigorous training and assessment to ensure comprehensive understanding of the survey methodology. Participation in this multicenter cross-sectional study was restricted to those who successfully passed the assessment. Prior to survey initiation, it was verified that each participating unit had been implementing ERAS perioperative protocols for a minimum of 2 years. The clinical teams in the participating hospitals, including anesthesiologists, nurses and surgeons, had received unified ERAS training.

Upon commencement of the survey, strict adherence to the predefined timeline was maintained. Following the acquisition of informed consent from subjects, patients undergoing surgery were selected based on predetermined inclusion criteria and subsequently sampled. From the initial medical education session, investigators meticulously recorded the implementation of each ERAS protocol component for every patient throughout the perioperative period. Each protocol should be approved by the patient prior to implementation (intraoperative protocols should also be approved by the patient prior to surgery). Concurrently, general patient information and other pertinent survey data points were collected.

Daily text message reminders were dispatched to prompt participants about the completion of rehabilitation logs, emphasizing aspects such as early mobilization and early oral intake. For instance, on the first postoperative day, in addition to preoperative education by nurses, mobile phone messages were utilized to remind patients of the requirement for out-of-bed activity for a duration of 2 h. The implementation of each ERAS protocol component for individual patients and subsequent outcomes were prospectively collected. For categorical elements, adherence was dichotomously marked as yes/no.

Furthermore, a specialized questionnaire was administered to each enrolled patient prior to surgery to assess their awareness, attitudes, and social environmental factors toward ERAS (the questionnaire is listed in Appendix 2). The questionnaire consisted of 4 sections and included 27 items in Chinese. Each item in this questionnaire was evaluated using a 5-point Likert scale (1 = Strongly Disagree, 2 = Disagree, 3 = Uncertain, 4 = Agree, 5 = Strongly Agree). The design of the questionnaire was based on the ERAS guidelines and previous related studies (14, 15), and the initial draft of the questionnaire was developed by a focus group consisting of 6 members engaged in ERAS research. They reached a consensus on the suitability of these 27 questions. It was further reviewed and revised by a multidisciplinary team (including 2 anesthesiologists, 2 surgeons, and 1 nurse). The experts reviewed each question and rated its importance on a 5-point scale [from 0 (not important) to 4 (very important)]. Then, the content validity index was calculated based on the proportion of experts who rated each question’s importance as level 3 or 4. Items with a content validity index < 0.80 were deleted or revised. A pre-survey was conducted on 20 hospitalized patients scheduled for abdominal surgery before the formal investigation. The final Cronbach’s coefficient was 0.862. The questionnaire was collected by the investigators, and the data were statistically analyzed by one person, then summarized by another person after verification. Questionnaires with obvious invalidity or randomly filled answers, such as those with more than 90% of identical options or those completed in less than 3 mins, were excluded.

### 2.3 Data collection

Adherence to each ERAS protocol component was thoroughly documented for all participants. Preoperative and postoperative patient ERAS adherence, including early ambulation, was ascertained through direct researcher observation. Each patient’s hospital stay was closely monitored. The adherence rate for individual patients was calculated as the ratio of fulfilled interventions to the total number of ERAS items (16). The mean total compliance was determined by averaging all perioperative ERAS interventions. ERAS adherence and inter-hospital variations across different grades were systematically evaluated and compared.

Stratified random sampling was employed, proportionate to the patient distribution across departments in each medical institution. Research data from each institution were uploaded to the platform system within 3 days of survey completion. The most significant factors influencing ERAS adherence were identified through a comprehensive analysis of completion rates for each ERAS protocol.

Patient data collected encompassed hospital affiliation, department, gender, age, educational background, and smoking history. Perioperative information, including surgical grade, history of abdominal surgery, ASA status, New York Heart Association (NYHA) cardiac function grading, nutritional status (assessed via Nutritional Risk Screening 2002), and postoperative complication grade (Clavien-Dindo Classification), was also documented. Furthermore, individual patient scores for each domain of the questionnaire were recorded.

### 2.4 Quality control

A designated experimenter in each medical institution was appointed as a quality control inspector by the project leader. These inspectors were tasked with conducting “primary quality control” for the ERAS adherence research. All quality control personnel had successfully completed relevant national clinical trial training and obtained Good Clinical Practice (GCP) training qualification certificates. Quality control checks were rigorously implemented, covering a minimum of 20% of the surveyed population daily, commencing from the onset of the survey.

### 2.5 Statistical analysis

In our preliminary survey, the standard deviation of ERAS adherence among patients in the southwestern region of China was 12.5%, with an allowable error margin set at 5%. The requirement of minimum sample size in this study was 602 participants to reach the statistical significance at two-sided 95% confidence interval. In addition, the total sample size was 753 by assuming a 20% attrition during follow-up. The sample size calculation was performed PASS 15.0 analysis program. Data entry was performed by two researchers using the double-entry method to ensure accuracy. Statistical analysis was conducted using SPSS 23.0 and GraphPad 8.0 software. Descriptive analysis was employed to characterize the study subjects. The Shapiro–Wilk test was utilized to examine the normal distribution of the data. In instances of clearly incorrect measurements or input errors, outliers were identified and eliminated through thorough investigation. For comparison of continuous variables between independent samples, either a t-test (parametric) or Mann–Whitney U test (non-parametric) was applied. When comparing more than two independent samples, One-Way ANOVA (parametric) or Kruskal-Wallis test (non-parametric) was utilized. Categorical variables were compared between groups using the chi-square test. For analyses involving more than two groups, pairwise comparisons were conducted using 2 × 2 chi-square tests when necessary, and the resulting *p* values were adjusted for multiple comparisons using the Bonferroni correction, with adjusted *p* values reported. The correlation between continuous variables was assessed using Pearson’s correlation coefficient (parametric) or Spearman’s rank correlation coefficient (non-parametric). The internal consistency of the questionnaires was evaluated using Cronbach’s ɑ. Multiple linear regression models were employed to assess the impact of various factors on ERAS adherence rates. GraphPad Prism 8 was used for the creation of graphs and charts. Statistical significance was set at *p* < 0.05 for all analyses.

## 3 Results

**Figure 1:**
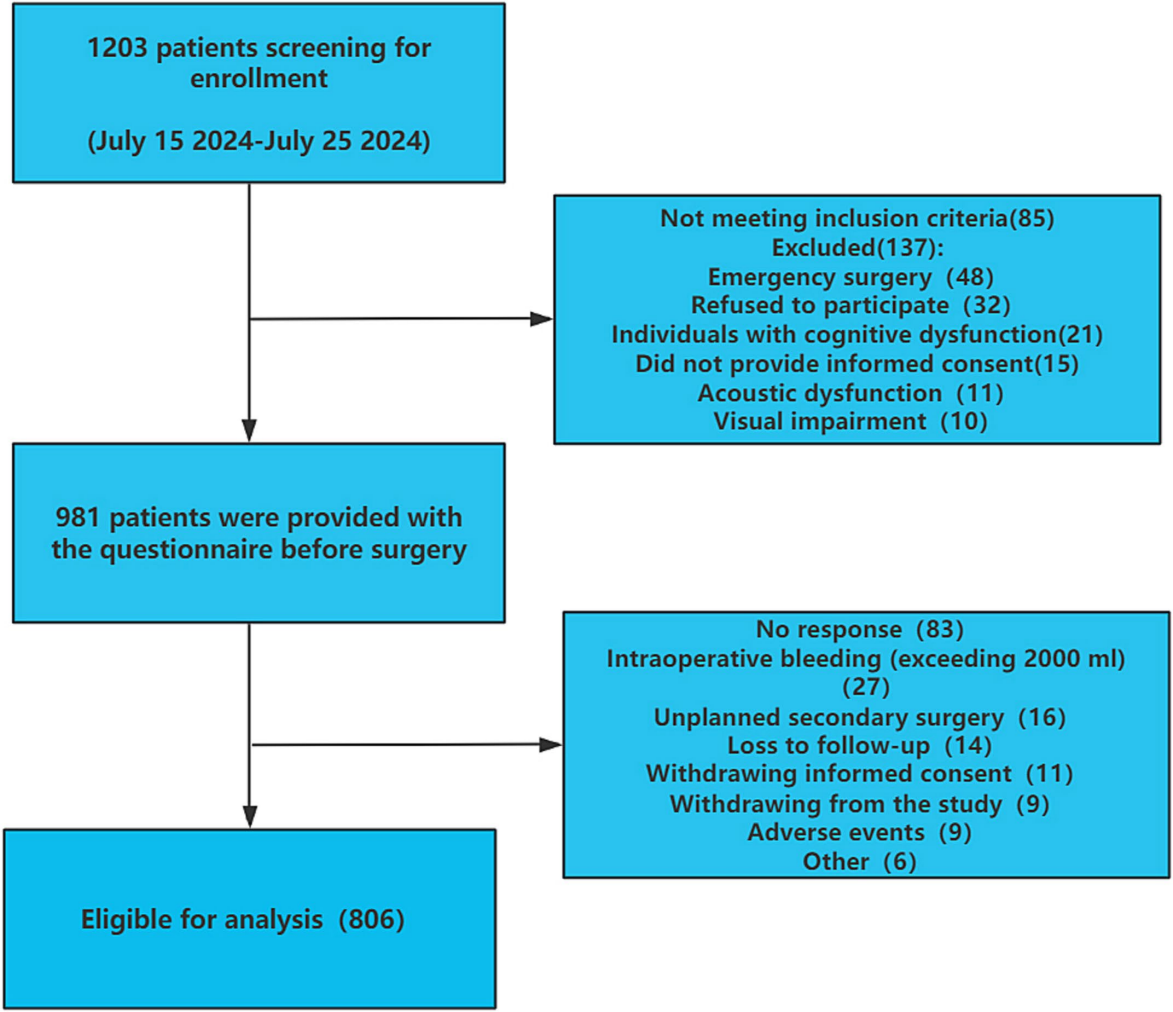
Flowchart of the study population selection.

A total of 1,203 patients were initially planned for observation across various medical centers in Southwestern China. However, 137 patients were excluded, and 85 patients did not meet the inclusion criteria. Questionnaires were distributed to the remaining 981 patients, resulting in 898 completed questionnaires. Finally, 806 patients were included in the analysis as 92 had dropped out due to intraoperative bleeding exceeding 2000 mL, unplanned secondary surgeries, loss to follow-up, withdrawal of informed consent, and other reasons as shown in Figure 1.

The 806 surgical patients ranged in age from 19 to 70 years, with a median age of 51 years. Male patients comprised 45.3% of the sample. Regarding educational background, 64.3% of patients had completed middle or high school, 16.4% had primary education or below, and 19.3% held college or university degrees. The distribution of surgery classifications revealed that the majority were classified as grade 3 to 4 surgeries, accounting for 29.8 and 29.9%, respectively. Preoperative ASA classifications were predominantly concentrated at levels I and II, representing 34.7 and 48.4% of patients, respectively. NYHA cardiac function classifications were approximately equally distributed between levels I and II. Additional general and surgical information, including hospital department, hospital grade, postoperative complications, abdominal surgery history, smoking history, and nutritional status, is also presented in Table 2.

**Table 2:** Baseline characteristics (N = 806).

Characteristics	*n*	%	ERAS compliance rate % (P25, P75)	*p* value
Hospital grade				<0.001
Tertiary hospital	445	55.3	68.8(56.3, 75.0)	
Non-tertiary center	361	44.7	68.8(56.3, 81.3)	
Age				0.933
18–35	159	19.7	68.8(56.3, 81.3)	
36–49	197	24.4	68.8(56.3, 81.3)	
50–59	228	28.3	68.8(56.3, 81.3)	
≥60	222	27.6	68.8(56.3, 81.3)	
Gender				0.569
Male	365	45.3	68.8(56.3, 81.3)	
Female	441	54.7	68.8(56.3, 81.3)	
Educational background				<0.001
Primary school or Below	132	16.4	62.5(56.3, 75.0)	
Junior High School	271	33.6	68.8(56.3, 75.0)	
High School/Vocational School	247	30.7	68.8(56.3, 81.3)	
College/University and Above	156	19.3	75.0(62.5, 93.8)	
Department				0.112
Gastroenterology	227	28.1	68.8(56.3, 87.5)	
Gynecology	198	24.5	68.8(56.3, 87.5)	
Hepatobiliary Surgery	187	23.2	68.8(56.3, 81.3)	
Urology	194	24.1	68.8(56.3, 87.5)	
Smoking history				0.132
Never smoked	412	51.1	68.8(56.3, 81.3)	
Occasional smoker	272	33.7	68.8(56.3, 75.0)	
Long-term smoker	122	15.2	68.8(56.3, 75.0)	
Surgical grade				<0.001
Grade 1	151	18.7	62.5(51.6, 73.4)	
Grade 2	174	21.6	65.6(56.3, 75.0)	
Grade 3	240	29.8	68.8(62.5, 81.3)	
Grade 4	241	29.9	68.8(68.8, 81.3)	
Abdominal surgery history				0.047
Yes	184	22.8	68.8(62.5, 81.3)	
No	622	77.1	68.8(56.3, 81.3)	
NYHA status				0.237
I	442	54.8	68.8(56.3, 81.3)	
II	364	45.1	68.8(56.3, 81.3)	
Nutritional status				0.353
NRS < 3	509	63.1	68.8(56.3, 81.3)	
NRS ≥ 3	297	36.9	68.8(56.3, 81.3)	
ASA status				0.177
I	280	34.7	65.6(56.3, 75.0)	
II	390	48.4	68.8(62.5, 81.3)	
III	136	16.9	68.8(62.5, 81.3)	
Postoperative complications				0.011
Grade 0, 1	584	72.5	68.8(56.3, 81.3)	
Grade 2	177	22.0	68.8(62.5, 81.3)	
Grade 3+	45	5.5	68.8(62.5, 87.5)	

ASA, American Society of Anesthesiologists; NYHA, New York Heart Association; NRS, Nutritional Risk Screening.

Significant differences in ERAS adherence rates were observed across various factors, including hospital grade (*p* < 0.001), educational background (*p* < 0.001), surgical grade (*p* < 0.001), abdominal surgery history (*p* = 0.047), and postoperative complications (*p* = 0.011).

**Figure 2:**
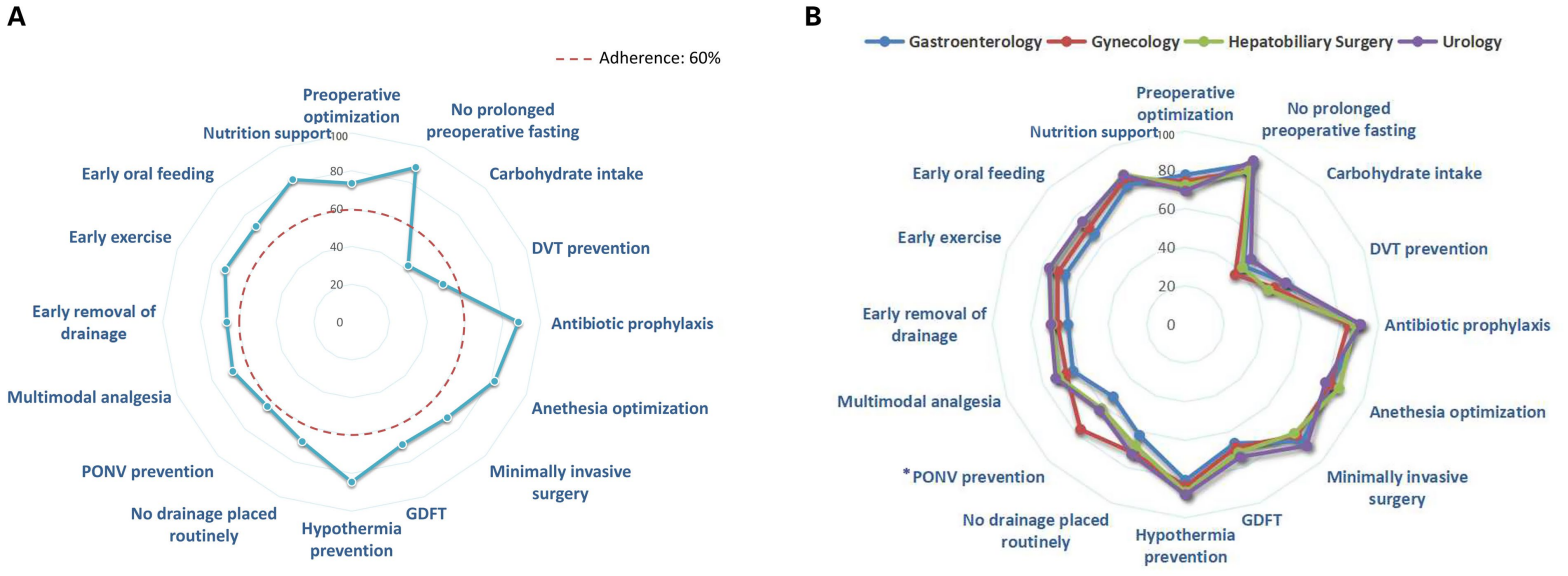
**(A)** Completion rate of individual ERAS component. **(B)** Completion rate of individual ERAS components in various departments. *The compliance rate of PONV prevention in gynecological patients was significantly higher than that in other departments, as determined by pairwise Chi-square comparisons with Bonferroni correction. Abbreviation: DVT, deep vein thrombosis; PONV, postoperative nausea and vomiting; GDFT, Goal-directed fluid therapy.

The average completion rate for the 16 ERAS protocols among the 806 surgical patients was 71.5% (56.3, 81.3%). The protocols with the highest completion rates were avoidance of prolonged fasting (88.6%), followed by prophylactic antibiotic use (88.2%). Conversely, the protocols with the lowest completion rates were preoperative oral carbohydrate intake (42.2%) and deep vein thrombosis prevention (52.4%), both of which had completion rates below 60% and require further improvement. Specific completion rates for each ERAS component are presented in Figure 2A. The completion rate of PONV prevention in gynecological patients was significantly higher than that in other departments, as shown in Figure 2B and Appendix Tables 3, 4.

**Figure 3:**
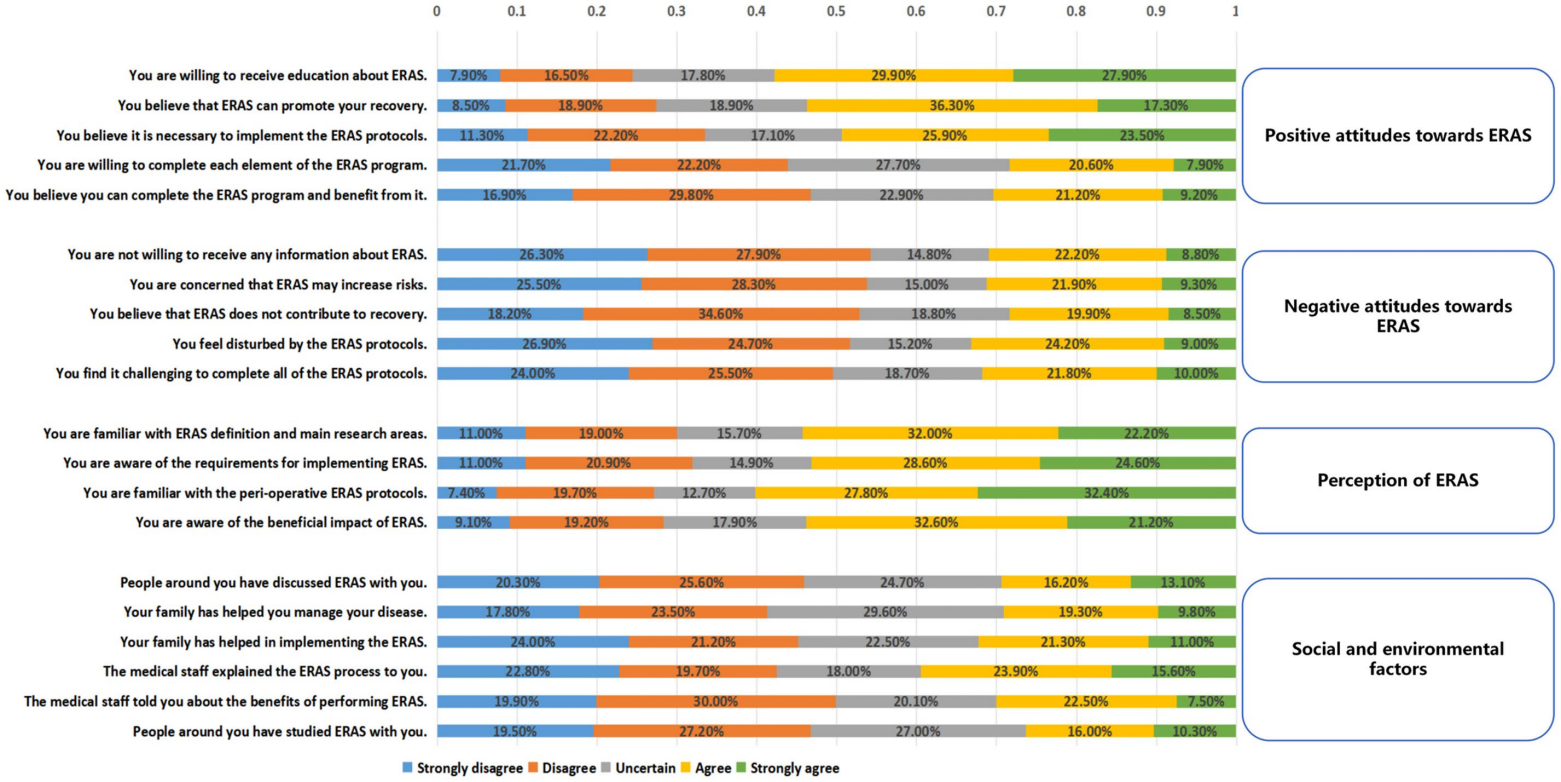
Inpatients’ positive attitudes toward ERAS (scores from 3 to 15), negative attitudes toward ERAS (scores from 3 to 15), reasons for positive attitudes toward ERAS (scores from 2 to 10), reasons for a negative attitudes toward ERAS (scores from 2 to 10), and degree of understanding of ERAS (scores from 3 to 15).

The questionnaire survey assessing patients’ perception, attitudes and social and environmental factors toward ERAS yielded an overall Cronbach’s *α* of 0.862. All patients had been educated about the ERAS upon admission. Concerning positive attitudes and motivations, a majority of patients (57.8%) report that they are willing to receive education about ERAS. Additionally, 53.6% of patients believe that ERAS can enhance their recovery process. Nevertheless, only 30.4% of the patients fully believed that they could complete the ERAS program (Figure 3).

Regarding negative attitudes and concerns, 31.2% of surveyed patients were concerned that ERAS might increase risks. Furthermore, 31.8% of the patients found it challenging to complete all of the ERAS protocols. The patients were familiar with the requirements for implementing ERAS (54.2%) and the beneficial impact of ERAS (53.8%). In this survey, social support from family, friends, or other patients was low (Figure 3).

**Figure 4:**
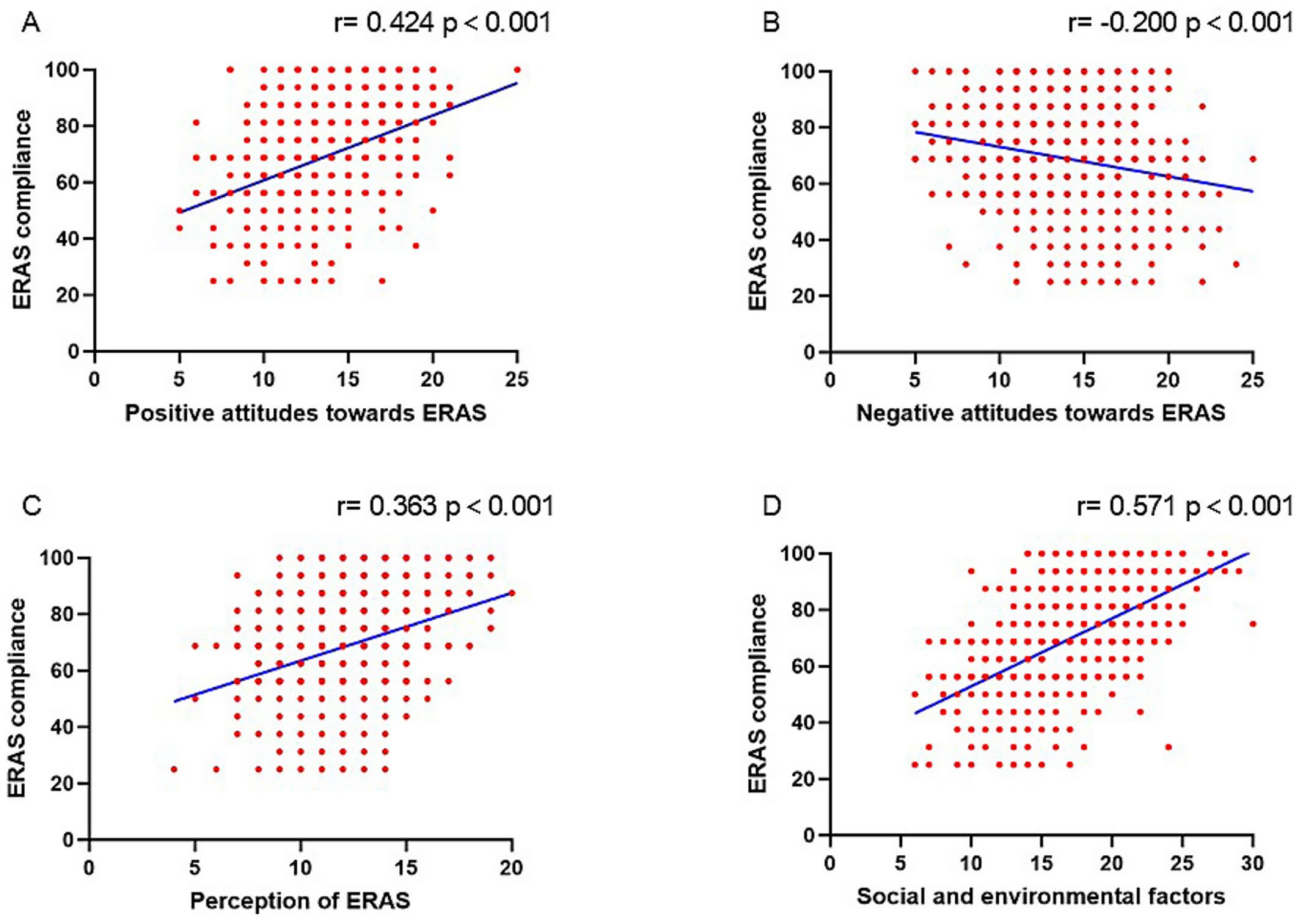
The correlation between ERAS adherence and positive attitudes toward ERAS **(A)**, negative attitudes toward ERAS **(B)**, perception of ERAS **(C)**, and social and environmental factors of ERAS **(D)**.

Figure 4 showed a higher ERAS adherence rate was associated with positive attitudes toward ERAS (*r* = 0.424, *p* < 0.001), perception of ERAS (*r* = 0.363, *p* < 0.001), social and environmental factors (*r* = 0.571, *p* < 0.001) and was negatively correlated with negative attitudes toward ERAS (*r* = − 0.200, *p* < 0.001). The variables which remained significantly associated with ERAS adherence rate on multivariable regression analysis included hospital grade (*p* = 0.039), educational background (College/University and above degree; *p* = 0.010), surgical grade 4 (*p* = 0.013), positive attitudes toward ERAS (*p* < 0.001), perception of ERAS (*p* < 0.001), negative attitudes toward ERAS (*p* = 0.002), and social and environmental factors (*p* < 0.001; Table 3).

**Table 3:** Multivariate liner regression analysis for ERAS compliance (N = 806).

Model	β	*SE*	β’	t	*p* value	95% CI for β	
						Lower bound	Upper bound
Non-tertiary center	−1.769	0.856	−0.052	−2.068	0.039	−3.448	−0.09
College/University and above (compared to primary school or below)	3.173	1.223	0.065	2.594	0.010	0.772	5.573
Surgical grade 4 (compared to 1)	2.398	0.968	0.063	2.477	0.013	0.498	4.298
Positive attitudes toward ERAS	1.399	0.147	0.247	9.51	<0.001	1.11	1.688
Negative attitudes toward ERAS	−0.630	0.124	−0.127	−5.073	0.002	−0.873	−0.386
Perception of ERAS	1.289	0.175	0.191	7.374	<0.001	0.946	1.632
Social and environmental support	1.824	0.112	0.433	16.248	<0.001	1.604	2.044

ERAS, Enhanced recovery after surgery.

## 4 Discussion

The southwestern region of China exhibits a significant disparity in medical resources compared to the developed eastern coastal areas. This disparity is manifested not only in the quantity of medical facilities but also in the quality and efficiency of medical services (16, 17). ERAS is a perioperative management model that necessitates a multidisciplinary, multi-faceted, and integrated approach to optimize patient outcomes (6, 18). Outcomes improve with increased compliance with recommended ERAS elements (19). Timely investigation, evaluation, and analysis of ERAS adherence rates are crucial for promoting improvement in ERAS adherence and facilitating the allocation of medical resources in these regions.

This study represents the first multicenter cross-sectional investigation of ERAS adherence rates and their influencing factors in Southwestern China. Based on ERAS guidelines and the characteristics of various medical centers in the region, 16 ERAS protocols were incorporated into perioperative management. The survey revealed an overall adherence rate of 71.5% (56.3, 81.3%) among 806 patients from 45 hospitals and 4 departments, which falls short of the ideal standard. The ERAS compliance rate observed in this study was comparable to those reported in two other recent investigations. A study on the implementation of ERAS for head and neck surgery reported an overall compliance rate of 62.6% (20). Another study demonstrated a compliance rate of 70% among patients with primary liver cancer undergoing hepatic resection (21). These findings suggest that an ERAS adherence rate exceeding 75% is considered acceptable, whereas a rate above 80% is required to effectively reduce postoperative complications. Furthermore, Gustafsson et al. (22) reported that in colorectal surgery, patients with an ERAS adherence rate of 70% or higher demonstrated a 42% lower risk of cancer-related death over 5 years compared to other patients. Although the results of this study approach this threshold, further improvement in ERAS adherence is required in Southwestern China.

Among the 16 ERAS protocols, 87.5% of the components exhibited an adherence rate of 60% or higher. However, significant variability in compliance was observed across different ERAS components. The protocols with the highest completion rates were avoidance of prolonged fasting (88.6%) and prophylactic antibiotic use (88.2%). Protocols with lower completion rates included preoperative oral carbohydrate intake (42.2%) and DVT prevention (52.4%). It is concerning that some medical staff continue to adhere to traditional fasting practices before surgery, failing to recognize that preoperative oral carbohydrate intake aids in maintaining normal gastrointestinal function and reducing preoperative insulin resistance (23). Additionally, concerns about bleeding risk among certain surgeons remain a significant barrier to the implementation of DVT prevention. In a Canadian study involving 2,876 patients, 20.1% of patients received all ERAS protocols, whereas in this study, the proportion was only 7.2% (24). The disparities between countries and regions are related to variations in medical resources and the knowledge and acceptance levels of ERAS among medical staff (25, 26). In further comparison of the completion rates of ERAS components in various departments, it was found that the completion rate of the PONV prevention for gynecology patients was significantly higher than that of other departments. This might be due to the higher incidence of PONV in gynecological surgery patients, thus prompting the medical team to pay more attention to it in their daily routines.

Previous studies have shown significant disparities in ERAS adherence rates across different levels of medical centers (27). Our study reveals that although the ERAS adherence rates of non-tertiary center and tertiary hospital share the same median, the overall difference is still statistically significant (*p* < 0.001), with tertiary hospital serving as a positive influencing factor for ERAS adherence (*p* = 0.046). This disparity can be attributed to the superior allocation of medical resources, recruitment of skilled personnel, and continuous medical knowledge updates in tertiary institutions, facilitating more effective implementation of ERAS programs. ERAS adherence rates demonstrated significant variation across different departments. Gastrointestinal surgery and gynecology surgery displayed higher adherence rates, likely due to the existence of well-established ERAS guidelines and a longer implementation history in these fields (5).

This study selected patients undergoing abdominal surgeries as research subjects because compared with neurosurgery and orthopedics, gastrointestinal and gynecological surgery departments have implemented ERAS earlier. This allows for a more in-depth analysis of the factors influencing ERAS adherence while minimizing potential research bias.

In China, surgeries are traditionally categorized into four levels based on complexity and risk. Our study demonstrates that Level 3 and Level 4 surgeries, when compared to Level 1 surgeries, positively impact ERAS adherence rates. A plausible explanation for this phenomenon is that more complex and higher-risk surgeries command greater attention from both medical staff and patients, resulting in increased willingness to implement every component of the ERAS protocols during the perioperative period.

In a prospective study (28), active ERAS protocols requiring direct patient involvement were identified as superior predictors of major postoperative morbidity and hospital stay duration compared to passive ERAS adherence rates. The costs associated with active ERAS protocols were significantly lower than those of passive protocols, emphasizing the importance of evaluating patient factors affecting ERAS adherence rates. Qin et al. (29) investigated the impact of health literacy on ERAS compliance among colon cancer patients, revealing that lower health literacy levels correlated with reduced ERAS adherence rates, extended hospital stays, and increased hospitalization costs. Lower health literacy levels were predominantly observed in individuals with lower educational attainment (30). The present results directly demonstrate the influence of patients’ educational backgrounds on ERAS adherence. Patients with a college/university degree and above exhibited a significantly higher median ERAS adherence rate compared to those with other educational backgrounds (75.0% vs. 68.8%). These highly educated patients demonstrated a superior ability to comprehend and adhere to perioperative recommendations provided by medical staff.

The questionnaire survey assessing patients’ understanding, attitudes, and social and environmental supports toward ERAS yielded an overall Cronbach’s *α* of 0.862, indicating high internal consistency among the items. The results suggest that more than half of the patients were familiar with ERAS, likely attributable to the ERAS education provided upon admission. The patient’s perception, attitude toward the ERAS protocols, as well as the support from the patient’s surrounding people (family, other patients or friends), have had a significant impact on the adherence rate of ERAS. Previous studies have demonstrated that patients with a positive attitude toward perioperative physical activity associated with ERAS are more likely to engage in early mobilization during their hospital stay (12, 31). However, the survey revealed that a considerable number of patients still exhibited skepticism or concern toward ERAS protocols. Similar to observations made by Wang et al. (32), common misconceptions among patients include the belief that postoperative rest, rather than early mobilization, is necessary for recovery, and that early postoperative feeding would overburden the intestines. Some patients expressed a need for more accurate and detailed explanations from medical professionals, perceiving that doctors and nurses provided instructions without adequate justification. These findings suggest that while admission education increased patients’ understanding of ERAS, it may not have effectively conveyed the specific benefits of each ERAS component. Consequently, to improve adherence rates, it may be necessary for a multidisciplinary team to collaboratively develop more comprehensive and accessible ERAS education materials, taking into account patient factors. Apart from the factors identified in this study, other research shows the barriers to the ERAS program related to clinical teams, including staff shortages (33), poor teamwork across departments, and resistance to abandoning outdated concepts (34).

Our study presents several limitations that warrant consideration. Firstly, although the investigation encompassed 16 commonly utilized ERAS protocols in Southwestern China, different hospitals in different areas may employ alternative ERAS protocols. This preference for different protocols could potentially result in findings that do not comprehensively represent the overall situation. The influencing factors on ERAS adherence in this study were investigated through patient-reported questionnaire responses. There may be reporting biases in the data, and further observation and randomized controlled studies will be useful in the future. Secondly, while our research examined numerous factors influencing ERAS adherence rates, the perceptions of the clinical team regarding ERAS were not explored. Notably, significant disparities exist in the understanding and attitudes toward ERAS among different doctors, which substantially impact the implementation of ERAS protocols (35). Thirdly, the general understanding and attitudes of patients toward ERAS may not be as favorable as depicted in our study. This discrepancy arises from the provision of ERAS-related education to each patient upon admission in our study, and this practice is not adopted by all hospitals worldwide.

## 5 Conclusion

The findings of this study indicate that ERAS adherence in China remains suboptimal. The research reveals that ERAS adherence rates are influenced by multiple factors, including hospital level, patients’ educational backgrounds, and surgical classification. Additionally, patients’ comprehension of ERAS, their attitudes and the support from the patient’s surrounding people toward the protocols play significant roles in determining adherence rates. To further enhance the implementation rate of ERAS, we recommend the following ways: strengthening the ERAS education for patients and their families, revising and updating the ERAS guidelines for each specific department, and ensuring that medical staff, patients and their families fully understand the ERAS protocols.

## Data Availability

The raw data supporting the conclusions of this article will be made available by the authors, without undue reservation.
