# Supplementary material for: Adherence to the enhanced recovery after surgery protocol and its influencing factors among patients in Southwestern China: a multicenter cross-sectional study
Source: Front Med (Lausanne). 2025 Oct 20;12:1660083. doi: 10.3389/fmed.2025.1660083 (PMC12580277; doi:10.3389/fmed.2025.1660083)
Supplement: Supplementary file 1 [file Table_1.DOCX]

**Appendix Table 3. The completion rate of individual ERAS components in various departments [N = 806, n(%) ]**

| **ERAS component** | **Gastroenterology**  **(n=227)** | **Gynecology**  **(n=198)** | **Hepatobiliary Surgery**  **(n=187)** | **Urology**  **（n=194）** | ***P* value** |
| --- | --- | --- | --- | --- | --- |
|  |  |  |  |  |  |
| Preoperative optimization | 176 (77.5%) | 147 (74.2%) | 135 (72.2%) | 134 (69.1%) | 0.255 |
| No prolonged preoperative fasting | 204 (89.9%) | 171 (86.4%) | 161 (86.1%) | 178 (91.8%) | 0.221 |
| Carbohydrate intake | 97 (42.7%) | 72 (36.4%) | 78 (41.7%) | 93 (47.9%) | 0.143 |
| DVT prevention | 128 (56.4%) | 99 (50.0%) | 87 (46.5%) | 108 (55.7%) | 0.150 |
| Antibiotic prophylaxis | 204 (89.9%) | 167 (84.3%) | 164 (87.7%) | 176 (90.7%) | 0.198 |
| Anethesia optimization | 185 (81.5%) | 161 (81.3%) | 161 (86.1%) | 152 (78.4%) | 0.272 |
| Minimally invasive surgery | 194 (85.5%) | 161 (81.3%) | 149 (79.7%) | 172 (88.7%) | 0.069 |
| GDFT | 151 (66.5%) | 136 (68.7%) | 134 (71.7%) | 144 (74.2%) | 0.338 |
| Hypothermia prevention | 183 (80.6%) | 166 (83.8%) | 162 (86.6%) | 171 (88.1%) | 0.150 |
| No drainage placed routinely | 141 (62.1%) | 142 (71.7%) | 127 (67.9%) | 141 (72.7%) | 0.078 |
| PONV prevention | 120 (52.9%) | 152 (76.8%) | 115 (61.5%) | 122 (62.9%) | ＜0.001 |
| Multimodal analgesia | 143 (63.0%) | 132 (66.7%) | 133 (71.1%) | 141 (72.7%) | 0.134 |
| Early removal of drainage | 138 (60.8%) | 131 (66.2%) | 129 (69.0%) | 135 (69.6%) | 0.203 |
| Early exercise | 153 (67.4%) | 141 (71.2%) | 142 (75.9%) | 148 (76.3%) | 0.131 |
| Early oral feeding | 151 (66.5%) | 140 (70.7%) | 140 (74.9%) | 146 (75.3%) | 0.157 |
| Nutrition support | 177 (78.0%) | 162 (81.8%) | 157 (84.0%) | 162 (83.5%) | 0.368 |

**Abbreviations:** DVT, Deep Vein Thrombosis; GDFT , Goal-Directed Fluid Therapy; PONV , Postoperative Nausea and Vomiting.
