# Supplementary material for: Adherence to the enhanced recovery after surgery protocol and its influencing factors among patients in Southwestern China: a multicenter cross-sectional study
Source: Front Med (Lausanne). 2025 Oct 20;12:1660083. doi: 10.3389/fmed.2025.1660083 (PMC12580277; doi:10.3389/fmed.2025.1660083)
Supplement: Supplementary file 2 [file Table_2.DOCX]

**Appendix Table 4. Pairwise Chi-square comparisons among departments regarding the completion rate of PONV prevention.**

| **Pairwise comparisons** | ***χ*²** | **Adjusted *P* value** |
| --- | --- | --- |
| **Gastroenterology** vs **Gynecology** | 25.201 | <0.001 |
| **Gastroenterology** vs **Hepatobiliary surgery** | 2.772 | 0.575 |
| **Gastroenterology** vs **Urology** | 3.899 | 0.290 |
| **Gynecology** vs **Hepatobiliary surgery** | 9.844 | 0.010 |
| **Gynecology** vs **Urology** | 8.326 | 0.023 |
| **Hepatobiliary surgery** vs **Urology** | 0.030 | 1.000 |

P values were adjusted using Bonferroni correction.
