# Supplementary material for: Adherence to the enhanced recovery after surgery protocol and its influencing factors among patients in Southwestern China: a multicenter cross-sectional study
Source: Front Med (Lausanne). 2025 Oct 20;12:1660083. doi: 10.3389/fmed.2025.1660083 (PMC12580277; doi:10.3389/fmed.2025.1660083)
Supplement: Supplementary file 3 [file Data_Sheet_1.DOCX]

**Appendix 1. The hospitals participating in the survey.**

| **The 21 tertiary hospitals** |
| --- |
| The First Affiliated Hospital of Chongqing Medical University of Chongqing |
| The University Town Hospital of Chongqing Medical University of Chongqing |
| The Second Affiliated Hospital of Chongqing Medical University of Chongqing |
| The Chongqing Traditional Chinese Medicine Hospital of Chongqing |
| The First People's Hospital of Chongqing |
| The Yongchuan Hospital of Chongqing Medical University of Chongqing |
| The Chongqing University Cancer Hospital of Chongqing |
| The Third People's Hospital of Chengdu of Sichuan Province |
| The Fourth People's Hospital of Chongqing |
| The First People's Hospital of Zunyi of Guizhou Province |
| The People's Hospital of Hechuan District of Chongqing |
| The People's Hospital of Qijiang District of Chongqing |
| The People’s Hospital of Liangping District of Chongqing |
| The People's Hospital of Kaizhou District of Chongqing |
| The Chongqing Armed Police Corps Hospital of Chongqing |
| The First People's Hospital of Liangjiang New Area of Chongqing |
| The Qianjiang Central Hospital of Chongqing |
| The People's Hospital of Jiulongpo District of Chongqing |
| The Ninth People's Hospital of Chongqing |
| The People's Hospital of Yubei District of Chongqing |
| The People's Hospital of Tongliang District of Chongqing |
| **The 24 non-tertiaty hospitals** |
| The Red Cross Hospital of Chongqing |
| The People’s Hospital of Hejiang County of Sichuan Province |
| The People’s Hospital of Jiangbei District of Chongqing |
| The People’s Hospital of Banan District of Chongqing |
| The People’s Hospital of Yunyang County of Chongqing |
| The People’s Hospital of Youyang County of Chongqing |
| The People’s Hospital of Fengjie County of Chongqing |
| The People’s Hospital of Yongchuan District of Chongqing |
| The People’s Hospital of Rongchang District of Chongqing |
| The People’s Hospital of Xiushan County of Chongqing |
| The Chenjiaqiao Central Hospital of Shapingba District of Chongqing |
| The People’s Hospital of Wuxi County of Chongqing |
| The People’s Hospital of Wulong District of Chongqing |
| The People’s Hospital of Wansheng Economic and Technological Development Zone of Chongqing |
| The People’s Hospital of Zhongxian of Chongqing |
| The Chongqing Construction Hospital of Chongqing |
| The People’s Hospital of Tongnan District of Chongqing |
| The Affiliated Hospital of Chongqing Three Gorges Medical College of Chongqing |
| The Southwest Aluminum Hospital of Chongqing |
| The People’s Hospital of Chengkou County of Chongqing |
| The People’s Hospital of Wushan County of Chongqing |
| The People’s Hospital of Pengshui County of Chongqing |
| The People’s Hospital of Fengdu County of Chongqing |
| The Fifth People’s Hospital of Wanzhou District of Chongqing |
