# Supplementary material for: Adherence to the enhanced recovery after surgery protocol and its influencing factors among patients in Southwestern China: a multicenter cross-sectional study
Source: Front Med (Lausanne). 2025 Oct 20;12:1660083. doi: 10.3389/fmed.2025.1660083 (PMC12580277; doi:10.3389/fmed.2025.1660083)
Supplement: Supplementary file 4 [file Data_Sheet_2.DOCX]

**Appendix 2. Questionaire**

| Dear Participants,  We, the researchers from the anesthesiology department of this hospital, cordially invite you to partake in our research study. The objective of this study is to comprehend patients' knowledge, attitudes, and behavioural intentions towards Enhanced Recovery after Surgery. This understanding will serve as a foundation for crafting evidence-based intervention strategies that could potentially improve the health conditions of numerous individuals in the future. Your participation in this study is entirely voluntary and has received ethical approval. Should you choose to take part, please review the following instructions.   1. Kindly complete the questionnaire. There are no right or wrong answers; you are simply required to provide responses based on your actual experiences. Should you have any queries during the answering process, please feel free to reach out to us. Once completed, please submit your responses in a timely manner. 2. This study involves a straightforward questionnaire survey that will not cause any physical or psychological harm. However, it will touch upon some privacy matters such as your gender and age. Rest assured, we will maintain strict confidentiality and not disclose your information. Please feel confident in providing your answers. 3. As a participant, you are entitled to stay informed about information related to this study and its progress. If you decide to withdraw from the study, please inform us. Your data will not be included in the study’s results.   Lastly, we extend heartfelt appreciation for sparing your valuable time to support our scientific research!  □ I have understood and agreed that the collected data will be used for scientific research.  Informed Consent Signature:  Date of Participation (Year/Month/Day): | | | | | | |
| --- | --- | --- | --- | --- | --- | --- |
| **Section One: Basic Information** | | | | | | |
| **1. Your Gender:** | a. Male | | | b. Female | | |
| **2. Your Age:** | Years Old | | | | | |
| 1. **Hospital:** |  | | | | | |
| 1. **Your hospitalization number:** |  | | | | | |
| 1. **Surgical department:** | a. Gastroenterology  b. Gynecology  c. Hepatobiliary Surgery  d. Urology | | | | | |
| **5. Your Level of Education:** | a. Primary School or Below  b. Junior High School  c. High School/Vocational School  d. College/University and Above | | | | | |
| **6. Do you have a smoking habit?** | a. Never Smoked  b. Occasional smoker  c. Long-term smoker | | | | | |
| **7. Have you ever had surgery on your abdomen?** | a. Yes  b. No | | | | | |
| **Section Two: Attitude towards ERAS** | | | | | | |
| 1. **You are willing to receive education about ERAS.** | a. Strongly　disagree | b. Disagree | c. Uncertain | | d.Agree | e. Strongly　agree |
| 1. **You believe that ERAS can promote your recovery.** | a. Strongly　disagree | b. Disagree | c. Uncertain | | d.Agree | e. Strongly　agree |
| 1. **You believe it is necessary to implement the ERAS protocols.** | a. Strongly　disagree | b. Disagree | c. Uncertain | | d.Agree | e. Strongly　agree |
| 1. **You are willing to complete each element of the ERAS program.** | a. Strongly　disagree | b. Disagree | c. Uncertain | | d.Agree | e. Strongly　agree |
| 1. **You believe you can complete the ERAS program and benefit from it.** | a. Strongly　disagree | b. Disagree | c. Uncertain | | d.Agree | e. Strongly　agree |
| 1. **You are not willing to receive any information about ERAS.** | a. Strongly　disagree | b. Disagree | c. Uncertain | | d.Agree | e. Strongly　agree |
| 1. **You are concerned that ERAS may increase risks.** | a. Strongly　disagree | b. Disagree | c. Uncertain | | d.Agree | e. Strongly　agree |
| 1. **You believe that ERAS does not contribute to recovery.** | a. Strongly　disagree | b. Disagree | c. Uncertain | | d.Agree | e. Strongly　agree |
| 1. **You feel disturbed by the ERAS protocols.** | a. Strongly　disagree | b. Disagree | c. Uncertain | | d.Agree | e. Strongly　agree |
| 1. **You find it challenging to complete all of the ERAS protocols.** | a. Strongly　disagree | b. Disagree | c. Uncertain | | d.Agree | e. Strongly　agree |
| **Section Three: Perception of ERAS** | | | | | | |
| 1. **You are familiar with ERAS definition and main research areas.** | a. Strongly　disagree | b. Disagree | c. Uncertain | | d.Agree | e. Strongly　agree |
| 1. **You are aware of the requirements for implementing ERAS.** | a. Strongly　disagree | b. Disagree | c. Uncertain | | d.Agree | e. Strongly　agree |
| 1. **You are familiar with the peri-operative ERAS protocols.** | a. Strongly　disagree | b. Disagree | c. Uncertain | | d.Agree | e. Strongly　agree |
| 1. **You are aware of the beneficial impact of ERAS.** | a. Strongly　disagree | b. Disagree | c. Uncertain | | d.Agree | e. Strongly　agree |
| **Section Four: Social and environmental factors** | | | | | | |
| 1. **People around you (family, other patients or friends) have discussed ERAS with you.** | a. Strongly　disagree | b. Disagree | c. Uncertain | | d.Agree | e. Strongly　agree |
| 1. **Your family has helped you manage your disease.** | a. Strongly　disagree | b. Disagree | c. Uncertain | | d.Agree | e. Strongly　agree |
| 1. **Your family has helped in implementing the ERAS.** | a. Strongly　disagree | b. Disagree | c. Uncertain | | d.Agree | e. Strongly　agree |
| 1. **The medical staff explained the ERAS process to you.** | a. Strongly　disagree | b. Disagree | c. Uncertain | | d.Agree | e. Strongly　agree |
| 1. **The medical staff told you about the benefits of performing ERAS** | a. Strongly　disagree | b. Disagree | c. Uncertain | | d.Agree | e. Strongly　agree |
| 1. **People around you have studied ERAS with you.** | a. Strongly　disagree | b. Disagree | c. Uncertain | | d.Agree | e. Strongly　agree |
